# Prevalence of Disordered Eating and Its Associated Factors From a Socioecological Approach Among a Sample of Spanish Adolescents: The EHDLA Study

**DOI:** 10.3389/ijph.2023.1605820

**Published:** 2023-03-27

**Authors:** José Francisco López-Gil, Estela Jiménez-López, Rubén Fernández-Rodríguez, Miram Garrido-Miguel, Desirée Victoria-Montesinos, Héctor Gutiérrez-Espinoza, Pedro J. Tárraga-López, Arthur Eumann Mesas

**Affiliations:** ^1^ Navarrabiomed, Hospital Universitario de Navarra (HUN), Universidad Pública de Navarra (UPNA), IdiSNA, Pamplona, Spain; ^2^ Department of Environmental Health, Harvard University T.H. Chan School of Public Health, Boston, MA, United States; ^3^ One Health Research Group, Universidad de Las Américas, Quito, Ecuador; ^4^ Health and Social Research Center, Universidad de Castilla-La Mancha, Cuenca, Spain; ^5^ Department of Nursing, Physiotherapy and Occupational Therapy, University of Castilla-La Mancha, Toledo, Spain; ^6^ Faculty of Pharmacy and Nutrition, UCAM Universidad Católica San Antonio de Murcia, Murcia, Spain; ^7^ Escuela de Fisioterapia, Universidad de las Américas, Quito, Ecuador; ^8^ Department of Medical Sciences, Faculty of Medicine, University of Castilla-La Mancha, Albacete, Spain; ^9^ Postgraduate Program in Public Health, Universidade Estadual de Londrina, Londrina, Brazil

**Keywords:** eating disorders, lifestyle, correlates, youths, obesity, overweight, sleep, immigrant status

## Abstract

**Objectives:** The aim of this study was twofold: a) to establish the prevalence of adolescents with disordered eating and b) to determine the factors associated with this prevalence in a sample of Spanish adolescents from the *Valle de Ricote* (Region of Murcia, Spain).

**Methods:** This cross-sectional study analyzed data from 730 adolescents (56.2% girls) from the EHDLA study. To determine the prevalence of disordered eating, the Sick, Control, One stone, Fat, Food (SCOFF) questionnaire was used. A socioecological approach was used to identify individual-, interpersonal-, or organizational-level factors associated with disordered eating.

**Results:** The prevalence of disordered eating was 30.1%. This condition was associated with female sex (odds ratio [OR] = 2.60; 95% confidence interval [CI], 1.81–3.73), immigrant status (OR = 2.22; 95% CI, 1.51–3.25), or excess weight (OR = 2.74; 95% CI, 1.93–3.89). Furthermore, for each additional hour slept, lower odds of having disordered eating were found (OR = 0.81; 95% CI, 0.67–0.98).

**Discussion:** Almost one-third of the sample of Spanish adolescents analyzed reported disordered eating. Female sex, immigrant status and excess weight are individual aspects that seem to be related to disordered eating among Spanish adolescents.

## Introduction

Eating disorders are severe and potentially life-threatening illnesses affecting people throughout the life span, with a specific influence on both the physical and psychological development of the young population (1, 2). Because of the serious consequences of untreated eating disorders (3), early identification and therapeutic intervention play a key role in minimizing their impacts. An increased prevalence of eating disorders in adolescents has been observed (4), which has spiked since 2020 due to the effects of the COVID-19 pandemic on eating habits and social behavior in this age group (5). Adolescence is an age phase that usually involves an increased risk of harmful behaviors that can have undesirable consequences for health status (6), such as eating disorders. However, although the epidemiology of eating disorders in European countries has been examined in adults (7, 8), an accurate estimate of the prevalence of these disorders in this risky age phase is still underaddressed in European countries.

The prevalence rate for eating disorders differs according to the population studied and the criteria applied to establish an eating disorder (9). A systematic review of prevalence studies (from 1994 to 2013) found broadly varied estimates in the lifetime prevalence of eating disorders, ranging from 0.3% to 0.6% for men and 1.0%–22.7% for women (10). Despite this, eating disorders are often poorly detected in the general population, resulting in delayed treatment (11). A cross-sectional study including more than 10,000 US adolescents aged 13–18 years reported lifetime prevalence estimates of anorexia nervosa, bulimia nervosa, and binge-eating disorder of 0.3%, 0.9%, and 1.6%, respectively (12). Moreover, the mean age of onset for eating disorders was 12.5 years (12).

In addition to diagnosed eating disorders, there are disordered eating, which can include weight-loss dieting, self-induced vomiting, self-induced vomiting, excessive exercise, binge eating, and use of diuretics or laxatives (13). Although disordered eating (e.g., unhealthy weight-control behaviors) predict outcomes linked with eating disorders (and obesity) in adolescents five years later (14), it is important to distinguish disordered eating from eating disorders (15). As such, these types of behaviors cannot be categorized as full-blown illnesses and should be carefully assessed since they may lead to eating disorders (13). In Spain, previous studies have described a prevalence of adolescents with disordered eating (measured by the Sick, Control, One stone, Fat, Food (SCOFF) questionnaire) ranging from 12% to 21% (16–18).

Specific causes of eating disorders have not been identified, but the potential etiology includes emotional and cognitive vulnerability, genetic predisposition, environmental and social factors, and weight stigma (19, 20). In relation to the factors associated with disordered eating, previous studies have shown associations with sex (21, 22), excess weight (18, 21–23), socioeconomic status (24), immigrant status (25, 26), screen time (23, 27), substance use (26), nutritional issues (28, 29), and family meals (30). However, most of these studies analyzed these relationships in isolation rather than including a broad spectrum of possible associated factors from a socioecological approach (31). This approach assumes that appropriate changes in the social environment will produce changes in individuals and that the support of individuals in the population is essential for implementing environmental changes (31). The socioecological approach may help to identify opportunities to prevent disordered eating by recognizing the individual (e.g., sex, attitudes), interpersonal (e.g., family, peers) and organizational (e.g., home, sports clubs) factors, among others, that could be associated with this condition.

Knowing the prevalence of disordered eating among adolescents and, especially, its associated factors could be useful to subsidize public policies and intervention programs for the prevention of these behaviors and, probably, of future eating disorders. Thus, the aim of this study was twofold: a) to establish the prevalence of adolescents with disordered eating and b) to determine the factors associated with this prevalence (i.e., age, sex, race/ethnicity, immigrant status, body mass index status, physical activity, sedentary behavior, sleep duration, muscle-strengthening activities, adherence to the Mediterranean diet, tobacco consumption, alcohol consumption, cannabis consumption, educational level of parents/guardians, socioeconomic status, type of schooling, area of residence, attending a sports club, dog owner, TV in bedroom, type of family, and number of siblings) in a representative sample of adolescents from the *Valle de Ricote* (Region of Murcia, Spain).

## Methods

### Study Design and Population

This cross-sectional study analyzed data from the EDHLA study, which included a representative sample of adolescents aged 12–17 years from the *Valle de Ricote* (Region of Murcia, Spain). All three secondary schools from this region (CE El Ope, IES Vicente Medina, and IES Pedro Guillén) were assessed for this study. Using a simple random sampling technique, the sample size estimated for the EDHLA study was 1,138. In this study, we only included adolescents with complete data for all the variables assessed. Thus, a total of 730 participants (56.2% girls) were included in this study. Data were collected during the 2021/2022 academic year. The detailed methodology of the EHDLA study has been published elsewhere (32).

To participate in this study, the parents or legal guardians of the adolescents received a signed written informed consent form before the participants’ enrollment. Additionally, both parents or legal guardians and their children received an information sheet explaining the aims of this research project and the tests and questionnaires administered. Likewise, adolescents were asked about their willingness to participate in the study.

This study was approved by the Bioethics Committee of the University of Murcia (ID 2218/2018) and the Ethics Committee of the Albacete University Hospital Complex and the Albacete Integrated Care Management (ID 2021-85). Moreover, the study was carried out following the Helsinki Declaration and respected the human rights of the participants enrolled.

### Procedures

#### Disordered Eating

To determine the prevalence of disordered eating, the SCOFF questionnaire was used (33). This tool contains five questions that can be self-administered with an acceptable sensitivity and specificity at a threshold of two (i.e., if patients provided positive responses to at least two of the five questions). In this study, the SCOFF was administered by two psychologists. The Spanish SCOFF questionnaire version has been validated for its use in primary care settings (34). A score ≥2 points was used to indicate disordered eating (34). For each specific eating disorder, this cutoff point showed a sensitivity and specificity (respectively) as follows: bulimia, 97.8% and 94.4%; anorexia, 93.1% and 94.4%; and eating disorders not otherwise specified, 100% and 94.4%.

### Associated Factors From a Socioecological Approach

#### Individual-Level Factors

Age, sex, and inmigrant status were self-reported by adolescents. Immigrant status was considered when adolescents met at least one of the following conditions: (a) immigrant parents, (b) born outside Spain, or (c) at least one parent from another country.

The Youth Activity Profile Physical (YAP), a 15-item self-report instrument, was used to obtain information related to physical activity and sedentary behavior among adolescents (35). The YAP is a self-administered 7-day recall (previous week) questionnaire adapted to young people aged 8–17 years. The items use a 5-point Likert scale and are separated into three sections: 1) activity at school, 2) activity out-of-school, and 3) sedentary habits. Physical activity (at school and out-of-school) and sedentary behavior (sedentary habits) scores were determined by summing the items in each section. Therefore, the total physical activity score (from item 1 to item 10) and sedentary behavior score (from item 11 to item 15) were averaged independently. The Spanish version of YAP (YAP-S) has been validated and adapted (36).

Sleep duration was assessed by asking participants for weekdays and weekend days separately: “What time do you usually go to bed?” and “What time do you usually get up?”. The average daily sleep duration was computed for each participant as follows: [(average nocturnal sleep duration on weekdays × 5) + (average nocturnal sleep duration on weekends × 2)]/7.

Concerning the quality diet, adherence to the Mediterranean diet was assessed by the Mediterranean Diet Quality Index for Children and Teenagers (KIDMED) index (37). The KIDMED index ranges from zero to 12 and is based on a 16-question test. Items reporting unhealthy characteristics related to the Mediterranean diet are scored with −1 point, and those reporting healthy characteristics are scored with +1 point. The sum of all scores from the KIDMED test will be used to categorize the scores into 3 different levels: (a) optimal Mediterranean diet (>8 points), (b) improvement needed to adjust intake to Mediterranean patterns (from 4 to 7 points), and (c) very low diet quality (≤3 points) (37).

Tobacco consumption was determined by the following question: “Have you ever smoked tobacco during the last 30 days?”. The response options were (a) never, (b) once or twice, (c) 3 to 5 times, (d) 6 to 9 times, (e) 10 to 19 times, (f) 20 to 29 times, or (g) 30 times or more (38). Subsequently, tobacco consumption was considered when participants smoked at least once or twice during the last 30 days. This same question was asked for alcohol and cannabis, and therefore, alcohol consumption and cannabis consumption were determined (38).

Following the standard protocols, the body weight of the adolescents was measured by an electronic scale (with an accuracy of 0.1 kg) (Tanita BC-545, Tokyo, Japan), while the height was determined by a portable height rod with an accuracy of 0.1 cm (Leicester Tanita HR 001, Tokyo, Japan). Body mass index was calculated by dividing body weight (in kg) by height (in squared meters). Furthermore, the body mass index z score was computed by the WHO age-specific and sex-specific thresholds (39)^,^ and therefore, body mass index status was computed: a) no excess weight (underweight/normal weight); b) excess weight (overweight/obesity).

#### Interpersonal Level Factors

Socioeconomic status (SES) was assessed with the Family Affluence Scale (FAS–III) (40). The FAS–III contains six different items related to vehicles, bedrooms, computers, bathrooms, dishwashers and travel and ranges from zero to 13 points. In addition, three different categories were determined: a) low SES (0–2 points); b) medium SES (3–5 points); and c) high SES (≥6 points). The greater the FAS–III, the higher the socioeconomic status.

In addition, adolescents were asked about the educational level of their father/mother/legal guardian individually. Possible choices will be (a) incomplete primary education, (b) complete primary education, (c) incomplete secondary education, (d) complete secondary education, (e) incomplete higher education, or (f) complete higher education.

#### Organizational Level Factors

The type of schooling was divided into two categories: (a) public and (b) private with public funds. Area of residence was divided into (a) urban (>5,000 inhabitants) and (b) rural (≤5,000 inhabitants) (41).

On the other hand, adolescents were asked several questions related to the home environment. Information about the number of siblings was solicited. Similarly, the type of family will be asked. The possible options were (a) nuclear family (including two-parent families, reconstituted/compound families, same-sex families, or adoptive families); (b) single-parent family (men or women); and (c) extended family (which includes not only the immediate family members as parents, children, and siblings but also grandparents, aunts, uncles, cousins, or other relatives). Adolescents were also asked if they owned a dog and, if so, if they walked the dog. Thus, participants will be assigned to one of three categories: nondog owner (those who do not own dogs as pets), unwalked dog owner (those who do not regularly walk his/her dog), and walked dog owner (those who regularly walk his/her dog as a hobby) (42). Furthermore, they were asked for the presence of TV in the bedroom (yes/no).

Finally, adolescents were asked for organized sport activities by the following question: “Do you attend a sport club?”. Response was dichotomic (i.e., yes/no).

### Statistical Analysis

Descriptive data were reported by means (*M*) and standard deviation (*SD*) (quantitative variables) or frequencies (*n*) and percentages (*%*) (qualitative variables). An *a priori* power analysis was conducted for sample size estimation according to Hsieh et al. (43). This estimation was determined based on the ability to detect small effects (OR = 1.44), considered to be small using Cohen’s criteria (44). A proportion of 30% of disordered eating was assumed (2, 45). With an alpha (*α*) value of 0.05 (type I error rate) and a beta value (*β*) of 0.20 (type II error rate), the minimum sample size needed with this effect size is N = 287 for binary logistic regression analysis. However, including additional explanatory variables in a regression model can increase the standard error of the coefficient estimate for the independent variables of interest and reduce statistical power. Hence, we used a coefficient of determination (*R* ^2^) value of 0.5 to account for a moderate amount of the variability in the outcome variable; therefore, the minimum sample size required was N = 573. Thus, the obtained sample size of N = 730 is above that is required to test the study hypothesis. Backward binary logistic regression analysis was performed with all the potential associated factors as independent variables and disordered eating (i.e., ≥2 points on SCOFF) as the dependent variable. Thus, the potential associated factors with the greatest *p*-value were excluded using a manual backward elimination approach (*p*-value for removal above 0.2) in each step of the binary logistic regression. A cutoff value of *p* < 0.2 has been suggested in some cases for variable selection in logistic regression, particularly when there are many potential predictors or the sample size is small (46). Moreover, the factors that remained in the last step with a *p* < 0.05 were selected as factors associated with disordered eating. Furthermore, we used model coefficients, omnibus tests, Nagelkerke’s *R* ^2^, and the Hosmer‒Lemeshow test to assess the performance of the logistic regression model in predicting the binary outcome variable. All analyses were performed with SPSS software (IBM Corp, Armonk, NY, USA) for Windows (version 28.0). Statistical significance was considered when the *p*-value was lower than 0.05.

## Results

Table 1 shows the descriptive information of the study participants globally and stratified by disordered eating. Participants included in this study were from CE El Ope (20.4%), IES Vicente Medina (53.5%), and IES Pedro Guillén (26.0%). The prevalence of disordered eating was 30.1% (95% CI 26.8–33.4). The prevalence of disordered eating was higher among adolescent girls, immigrants, and those with excess weight (*p* < 0.001 for all). Furthermore, the number of Caucasian participants was lower in the disordered eating group (*p* = 0.002). A greater number of adolescents reporting cannabis use was found in those with disordered eating (*p* = 0.022). Last, a lower number of adolescents with parents/legal guardians with university studies was found in those with disordered eating (*p* < 0.05 for all).

**TABLE 1:** Descriptive data of the study participants (The Eating Healthy and Daily Life Activities (EHDLA) study, *Valle de Ricote*, Region of Murcia, Spain. 2023).

Variables	Total sample (N = 730; 100%)	Non-disordered eating (*n* = 510; 69.9%)	Disordered eating (*n* = 220; 30.1%)	*p*
	M ± SD/n (%)	M ± SD/n (%)	M ± SD/n (%)	
Individual level				
Age (years)	13.9 ± 1.5	13.9 ± 1.5	14.0 ± 1.6	0.400
Sex (%, girls)	410 (56.2)	259 (50.8)	151 (68.6)	**<0.001**
Race/Ethnicity (%, Caucasian)	617 (84.5)	445 (87.3)	172 (78.2)	**0.002**
Immigrant status (%, native)	555 (76.0)	411 (80.6)	144 (65.5)	**<0.001**
BMI status (%, excess weight^a^)	342 (46.7)	207 (40.6)	135 (61.4)	**<0.001**
YAP-S physical activity (score^b^)	2.6 ± 0.7	2.6 ± 0.7	2.6 ± 0.7	0.293
YAP-S sedentary behavior (score^c^)	2.6 ± 0.6	2.6 ± 0.7	2.6 ± 0.7	0.639
Sleep duration (hours)	8.2 ± 0.9	8.3 ± 0.9	8.1 ± 1.0	**0.010**
Muscle-strengthening activities (days)	2.4 ± 1.9	2.4 ± 1.9	2.3 ± 1.9	0.690
KIDMED (score)	6.5 ± 2.5	6.5 ± 2.4	6.5 ± 2.6	0.902
Adherence to the MD				
Low MD	90 (12.3)	58 (11.4)	32 (14.5)	
Moderate MD	370 (50.5)	266 (52.2)	103 (46.8)	0.314
High MD	272 (37.2)	186 (36.5)	85 (38.6)	
Tobacco consumption (%, yes)	51 (7.0)	30 (5.9)	21 (9.5)	0.075
Alcohol consumption (%, yes)	131 (17.9)	85 (16.7)	46 (20.9)	0.170
Cannabis consumption (%, yes)	44 (6.0)	24 (4.7)	20 (9.1)	**0.022**
*Interpersonal level*				
Educational level (father) (%, university studies)	160 (21.9)	125 (24.5)	35 (15.9)	**0.010**
Educational level (mother) (%, university studies)	208 (28.5)	160 (31.4)	48 (21.8)	**0.009**
FAS-III (score)	8.1 ± 2.1	8.2 ± 2.1	7.9 ± 2.2	**0.043**
Low SES	161 (22.1)	104 (20.4)	57 (25.9)	
Medium SES	388 (53.2)	273 (53.5)	115 (52.3)	0.193
High SES	181 (24.8)	133 (26.1)	48 (21.8)	
*Organizational level*				
Type of schooling (%, public)	581 (79.6)	402 (78.8)	179 (81.4)	0.435
Area of residence (%, urban)	541 (74.1)	380 (74.5)	161 (73.3)	0.707
Attending in a sports club (%, yes)	250 (34.5)	182 (35.7)	66 (30.0)	0.137
Dog owner				
Nondog owner	459 (62.7)	312 (61.2)	147 (66.8)	
Unwalked dog owner	55 (7.5)	38 (7.5)	17 (7.7)	0.271
Walked dog owner	216 (29.6)	160 (31.4)	56 (25.5)	
TV in bedroom (%, yes)	312 (42.7)	221 (43.3)	91 (41.4)	0.622
Type of family				
Extended family	20 (2.7)	12 (2.4)	8 (3.6)	
Single-parent family	38 (5.2)	31 (6.1)	7 (3.2)	0.179
Nuclear family	672 (92.1)	467 (91.6)	205 (93.2)	
Siblings (number)	0.9 ± 0.3	0.9 ± 0.3	0.9 ± 0.3	0.240

^a^ Prevalence of excess weight according to the World Health Organization criteria.

^b^ Average of items 1 to 10 of the YAP-S questionnaire.

^c^ Average of items 11 to 15 of the YAP-S questionnaire. BMI, body mass index; FAS-III, Family Affluence Scale–III; KIDMED, Mediterranean Diet Quality Index in children and adolescents; MD, Mediterranean diet; SES, socioeconomic status; TV, television; YAP-S, Spanish Youth Activity Profile. Bold indicates a *p*-value <0.05.

**FIGURE 1:**
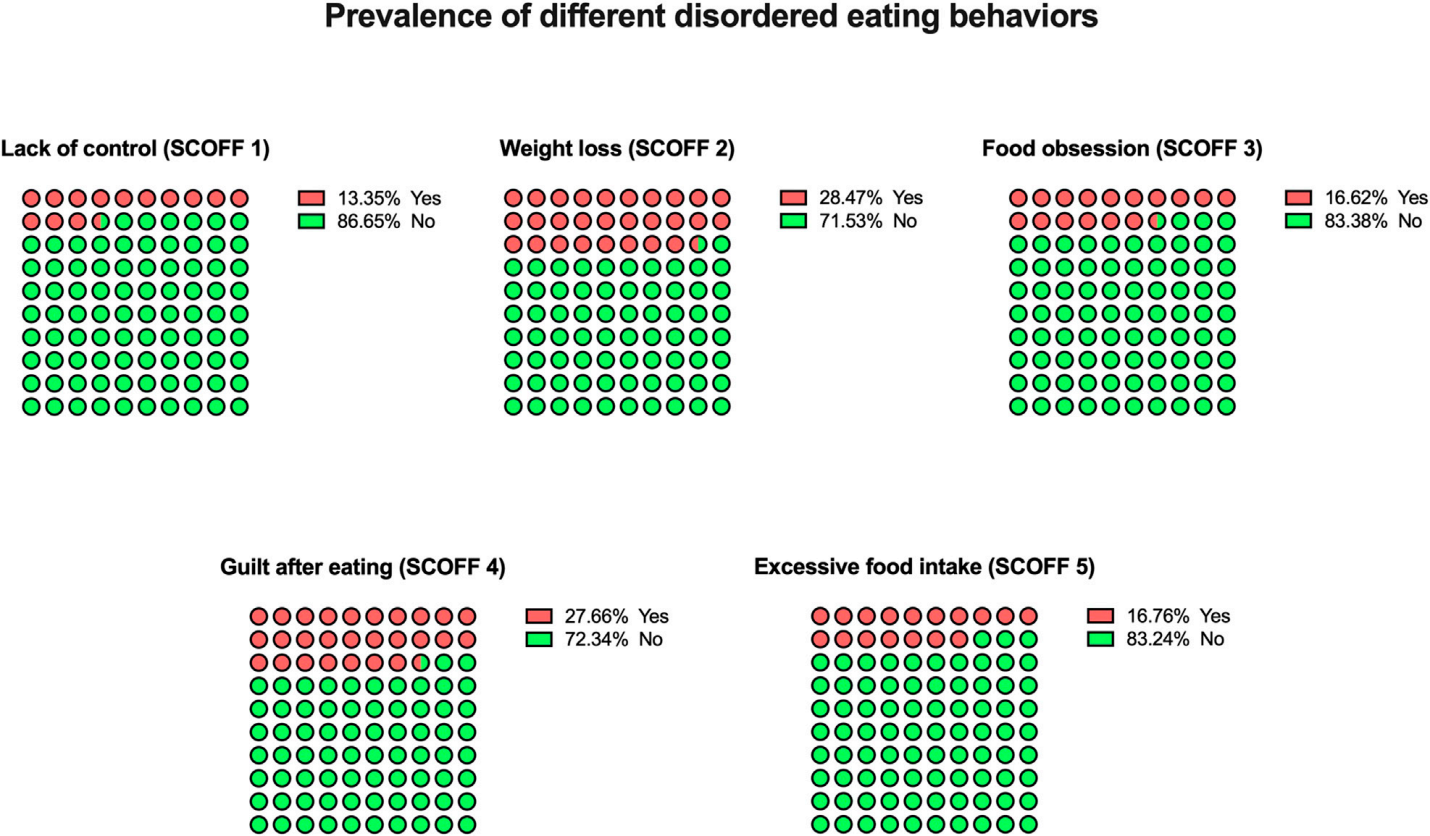
Prevalence of different disordered eating behaviors among adolescents included in the Sick, Control, One stone, Fat, Food questionnaire (The Eating Healthy and Daily Life Activities (EHDLA) study, *Valle de Ricote*, Region of Murcia, Spain. 2023). SCOFF, Sick, Control, One stone, Fat, Food.

Figure 1 displays the prevalence of the different behaviors evaluated in the SCOFF questionnaire among the sample of adolescents analyzed. The disordered eating behavior most reported was related to “weight loss” (28.5%). Conversely, “lack of control” was the lowest disordered eating behavior reported by adolescents (13.4%).

Table 2 describes the associated factors maintained in the last step of the binary logistic regression model. Backward logistic regression analysis included 16 steps, in which the least statistically significant correlation was removed from the model in each step. Female sex (OR = 2.60; 95% CI, 1.81–3.73), immigrant status (OR = 2.22; 95% CI, 1.51–3.25) and excess weight (OR = 2.74; 95% CI, 1.93–3.89) were associated with disordered eating. Furthermore, for each additional hour slept, the odds of having disordered eating were lower (OR = 0.81; 95% CI, 0.67–0.98).

**TABLE 2:** Binary logistic regression analysis with potential associated factors as independent variables and disordered eating behaviors as a dependent variable among Spanish adolescents (The Eating Healthy and Daily Life Activities (EHDLA) study, *Valle de Ricote*, Region of Murcia, Spain. 2023).

Predictors	OR^a^		95% CI	P
Individual level				
Age	Variable excluded in step 9^b^			
Sex				
Boys	1			
Girls	**2.60**		**1.81–3.73**	**<0.001**
Race/Ethnicity	Variable excluded in step 5 ^b^			
Immigrant status				
Native	1			
Immigrant	**2.22**		**1.51–3.25**	**<0.001**
BMI status ^c^				
No excess weight	1			
Excess weight	**2.74**		**1.93–3.89**	**<0.001**
YAP-S physical activity (per 1 point)	1.23		0.95–1.59	0.125
YAP-S sedentary behavior	Variable excluded in step 11 ^b^			
Sleep duration (per 1 hour)	**0.81**		**0.67–0.98**	**0.033**
Muscle-strengthening activities	Variable excluded in step 4 ^b^			
Adherence to the MD	Variable excluded in step 12 ^b^			
Tobacco consumption	Variable excluded in step 7 ^b^			
Alcohol consumption	Variable excluded in step 6 ^b^			
Cannabis consumption	Variable excluded in step 15 ^b^			
No	1			
Yes	1.87		0.95–3.70	0.072
Interpersonal level				
Educational level (father)				
No university studies	1			
University studies	0.65		0.42–1.01	0.055
Educational level (mother)	Variable excluded in step 1 ^b^			
FAS-III status	Variable excluded in step 2 ^b^			
Organizational level				
Type of schooling	Variable excluded in step 3 ^b^			
Area of residence	Variable excluded in step 8 ^b^			
Attending in a sports club	Variable excluded in step 9 ^b^			
Dog owner status	Variable excluded in step 13 ^b^			
TV in bedroom	Variable excluded in step 10 ^b^			
Type of family				
Extended family	1			
Single-parent family	0.29		0.08–1.01	0.062
Nuclear family	0.71		0.27–1.87	0.489
Number of siblings	Variable excluded in step 14 ^b^			
Model coefficient omnibus tests: *χ* ^2^ = 98.335; *df* = 10; *p* < 0.001				
Model summary: Nagelkerke’s *R* ^2^ = 0.179				
Hosmer‒Lemeshow test: *χ* ^2^ = *7.095; df = 8; p* = 0.526				

^a^ Non-disordered eating selected as a reference group.

^b^ Logistic regression models with the backward stepwise variable selection method (statistical criterion *p* > 0.20). BMI, body mass index; df, degrees of freedom; FAS, Family Affluence Scale; MD, Mediterranean diet; TV, television; YAP-S, Spanish Youth Activity Profile. Bold indicates associated factors with a p-value < 0.05.

^c^ Prevalence of excess weight according to the World Health Organization criteria.

## Discussion

Overall, our findings showed that three out of 10 participants reported disordered eating. This result is slightly higher than previous studies performed in Spain, including samples of adolescents (17, 18, 47). The highest disordered eating behavior reported by adolescents was related to weight loss. One possible explanation for this greater prevalence is that excess weight and disordered eating behaviors usually coexist (48). Given that the Region of Murcia has been identified as the region of Spain with the highest prevalence of excess weight in young people (49) and the high prevalence of excess weight found in our study, this finding seems to be justified. Furthermore, in the bivariate analyses, the prevalence of disordered eating was significantly different according to sex, race, immigration status, BMI status, sleep duration, cannabis use, FAS–III (score) or educational level (parents/legal guardians). Notwithstanding, when a socioecological approach was applied, some individual-level factors, such as sex, immigrant status, excess weight and sleep duration, were significantly associated with disordered eating, which suggests that fundamental disparities exist in relation to this worrisome condition among the sample of Spanish adolescents analyzed.

Our findings indicated that female sex was linked with disordered eating. This result agrees with previous studies (2, 21, 22) but disagrees with others (23, 47). Although sex differences in disordered eating are relatively minor in adolescence (50), these disorders are known to predominate among girls (2, 51). Traditionally, studies have focused predominantly on the female sex, but currently, this has no longer been considered a girl-specific issue (51). Similarly, eating disorders and disordered eating in boys may present differently than in girls, specifically with muscle-oriented disordered eating (52). The reasons for sex differences in prevalence are not well known (50). It has been noted that disordered eating are frequently unobserved among boys (53). Boys are presumed to underreport the problem because of the stigma of female sex eating disorders (54), and disordered eating are considered specific to girls/women (53). Additionally, it has been advised that the current diagnostic criteria of eating disorder (55) fail to detect disordered eating behaviors unique to boys (i.e., behaviors aimed at increasing muscle mass and weight to counteract body image dissatisfaction (53)).

Our results also indicated that immigrant status was associated with disordered eating. This result is in line with a previous longitudinal study in Spain carried out by Esteban-Gonzalo et al. (25), who found in adolescents that immigrant populations, mainly boys, seem to be more susceptible to disordered eating than non-immigrant boys. Similarly, Hölling and Schlack (26) found that the prevalence of German adolescents with disordered eating was approximately 50% higher among immigrants than among non-immigrants. In fact, our understanding of and capacity to treat eating disorders among ethnic and racial minority groups continues to improve (56). One possible reason explaining this result could lie in the acculturative stress and ethnic minority position, since most of the evidence suggests a substantial link between this condition and eating disorder psychopathology (57). Another possible justification is that marriages including a foreign-born and a native-born partner could lead to greater levels of unsolved family agreements, a variable associated with increased disordered eating (58). However, why marriage including a foreign-born and a native-born partner is related to increased disordered eating for offspring is still an open question (59).

On the other hand, we found that excess weight was linked with disordered eating. This result matches the scientific literature (2 López-Gil et al.). For instance, a previous study by D’Anna et al. (21), reported that the prevalence of disordered eating among Italian adolescents was greater in the higher BMI categories. Similarly, Feng and Abebe (23) found that perceived excess weight was associated with disordered eating in their study among Chinese adolescents. Gutiérrez et al. (22) also found that a higher body mass index was linked with disordered eating. There are some potential reasons for this result. For instance, adolescents who have excess weight may adopt disordered eating behaviors while trying to lose weight, and early efforts to lose weight by eating healthy may progress to skipping meals, severe dietary restriction, prolonged periods of starvation, the use of laxatives, diet pills, or self-induced vomiting (60). In addition, as previously mentioned, excess weight and disordered eating frequently overlap (48). Thus, the high prevalence of excess weight found in the sample of Spanish adolescents analyzed could explain this result. In accordance with this finding, the need to consider eating disorders and excess weight as part of a continuum of weight-related disorders has been highlighted (61, 62).

Another interesting finding was that sleep duration was inversely linked with disordered eating. It has been suggested that disturbed sleep is a frequent complaint among individuals with a broad range of psychopathology and may be a particularly important factor in the development and course of eating disorders in adolescence (63). However, the literature on sleep and disordered eating is still scarce. Supporting this idea, variations in sleep duration may affect the susceptibility to losing control over eating after experiencing negative affect in a young population, and variations in sleep duration may impact susceptibility to losing control over eating after experiencing negative feelings in a young population (64). Furthermore, chronic sleep restriction may increase the likelihood of suboptimal dietary behavior in adolescents with excess weight (65). This is because they do not experience an increased inhibition-related neural response to counter the potential increase in reward-related neural response after sleep restriction (i.e., neural processes may be deficient in neutralizing increases in reward responding to mitigate excessive food intake) (65).

The present study has certain limitations that must be acknowledged. First, because of the cross-sectional nature of this study, a causal relationship cannot be established. Second, due to the inclusion of binge eating disorder and other specified eating disorders in the DSM-5, there is not enough evidence to support the use of SCOFF in primary care and community-based settings for screening the entire range of eating disorders. However, a meta-analysis by Kutz et al. (66) including 25 validation studies demonstrated that the SCOFF is a useful and simple screening tool for some of the most prevalent eating disorders (i.e., bulimia nervosa, anorexia nervosa). Third, we used self-report questionnaires to assess disordered eating and consequently, both social desirability and recall bias are possible. Conversely, this study has some strengths. For instance, we included a representative sample of adolescents from *Valle de Ricote* (Spain), which provides substantial external validity to our results. Similarly, these findings offer additional cross-sectional evidence of several understudied factors associated with disordered eating among adolescents from a socioecological approach.

In conclusion, almost one-third of Spanish adolescents from the *Valle de Ricote* (Region of Murcia, Spain) showed disordered eating, which could severely influence their general health. The associations found according to female sex, immigrant status and excess weight underline that there are fundamental individual disparities in having disordered eating among Spanish adolescents. Furthermore, these results suggest that longer sleep duration could help prevent disordered eating. Our findings point to the relevance of providing specific intervention programs to decrease the prevalence of disordered eating, mainly for adolescent girls, immigrants, and those with excess weight.
